# Pyrrolo‐Fused Phenanthridines as Potential Anticancer Agents: Synthesis, Prediction, and Biological Evaluation

**DOI:** 10.1002/jbt.70443

**Published:** 2025-08-13

**Authors:** Ashraf Al‐Matarneh, Natalia Simionescu, Alina Nicolescu, Narcis Cibotariu, Ramona Danac, Maria‐Cristina Al‐Matarneh, Ionel I. Mangalagiu

**Affiliations:** ^1^ Faculty of Chemistry Alexandru Ioan Cuza University of Iasi Iasi Romania; ^2^ Centre of Advanced Research in Bionanoconjugates and Biopolymers “Petru Poni” Institute of Macromolecular Chemistry of Romanian Academy Iasi Romania; ^3^ NMR Laboratory “Petru Poni” Institute of Macromolecular Chemistry of Romanian Academy Iasi Romania

**Keywords:** ADME, anticancer, phenanthridine, pyrrole

## Abstract

We report the synthesis of four novel monoquaternary salts and four fused pyrrolo‐phenanthridine compounds, fully characterized by NMR, FT‐IR, and mass spectrometry. Guided by theoretical predictions, including molecular docking studies, we assessed their cytotoxic activity and biocompatibility. The docking results revealed notably stronger binding affinities compared to Phenstatin, a known anticancer agent, suggesting high therapeutic promise. In vitro cytotoxicity was evaluated on osteosarcoma cell lines HOS and MG‐63, showing a marked cell‐line‐dependent response: all compounds inhibited MG‐63 cell viability by approximately 50%, while their effect on HOS cells was more modest (20%–30%). No significant activity was observed against the MeWo melanoma line. Nonetheless, compounds **3a–d**, **5a**, and **5b** demonstrated good biocompatibility at 10 and 50 µM and selective cytotoxicity toward MG‐63 cells. These findings, combined with favorable docking profiles, highlight the potential of these compounds as anticancer candidates and justify further investigation.

## Graphical abstract

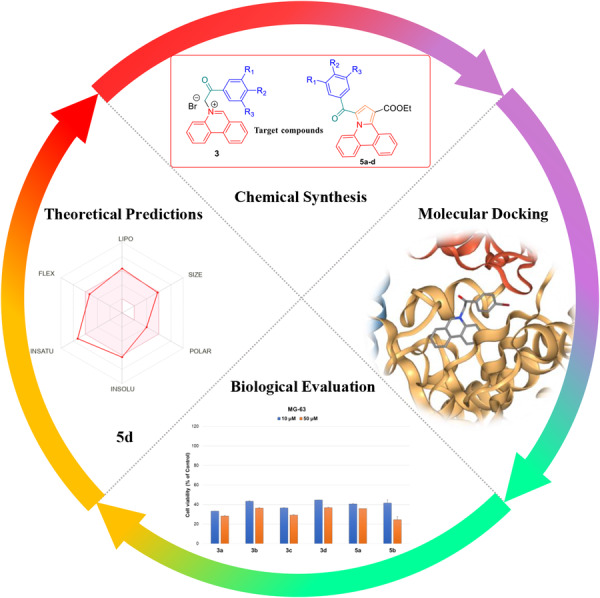

## 1 Introduction

**Figure 1:**
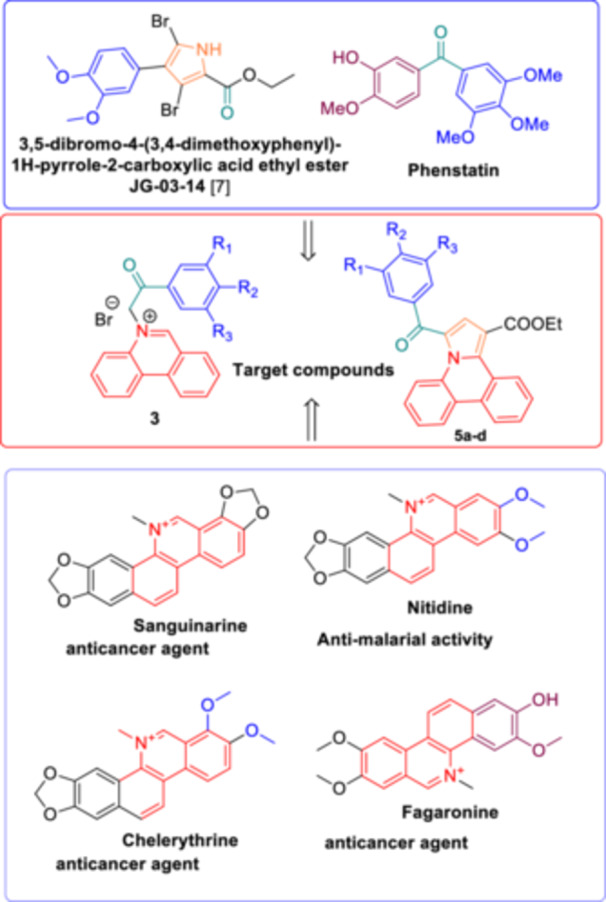
Molecular structures and design of biological active phenanthridine based structures.

Over 80 alkaloids, such as Sanguinarine [1], Fagaronine, Nitidine, or Chelerythrine (Figure 1) featuring the benzo[*c*]phenanthridine scaffold have been discovered, isolated from plants and studied for their biological properties particularly in cancer therapy and antimicrobial treatments [2]. The phenanthridine scaffold serves as a crucial synthetic motif commonly found in biologically active compounds with antimicrobial, antifungal, anti‐inflammatory, antiproliferative, and optoelectronic properties. Moreover, some of its derivatives are utilized as antiprotozoal, antibacterial, and anticancer agents [3].

Conversely, pyrrole derivatives have garnered significant attention in medicinal chemistry due to their potent antitumor and antimicrobial properties [4]. These compounds exhibit a broad spectrum of biological activities, including anticancer [5], anti‐inflammatory, antiviral, and antibacterial effects [6]. The versatility of the pyrrole scaffold allows for the design of molecules that can interact with various biological targets, making them valuable candidates in drug development [7, 8].

In the realm of anticancer research, pyrrole‐based compounds have demonstrated the ability to inhibit key enzymes [9, 10] and pathways involved in tumor growth and proliferation [11]. For instance, certain pyrrole derivatives have been identified as inhibitors of topoisomerase I and II, enzymes crucial for DNA replication and transcription in cancer cells [12]. By interfering with these enzymes, pyrrole compounds can induce apoptosis and suppress tumor progression [13].

One such target is tubulin, a structural protein integral to microtubule formation, which plays a crucial role in cell division. Pyrrole‐based compounds have been identified as potent inhibitors of tubulin polymerization, leading to cell cycle arrest and apoptosis in cancer cells [14]. For instance, the compound 3,5‐dibromo‐4‐(3,4‐dimethoxyphenyl)‐1H‐pyrrole‐2‐carboxylic acid ethyl ester (JG‐03‐14) (Figure 1) has demonstrated significant microtubule depolymerizing activity. JG‐03‐14 binds to the colchicine‐binding site on tubulin, disrupting microtubule dynamics, causing mitotic arrest, and subsequently inducing apoptosis in various cancer cell lines. Notably, this compound has shown efficacy against multidrug‐resistant cancer cells, highlighting its potential as a valuable chemotherapeutic agent [15].

Building on these insights, our study focuses on the synthesis and evaluation of novel Phenstatin analogues (Figure 1) incorporating substituted pyrrolophenanthridines as key structural components. By integrating the cytotoxic potential of phenanthridine and pyrrole derivatives with the established antitumor activity of Phenstatin, we aim to explore their structure–activity relationships and assess their therapeutic potential. This study contributes to the ongoing development of innovative anticancer agents with improved selectivity and efficacy, paving the way for future advancements in targeted chemotherapy [9, 16, 17, 18, 19, 20, 21, 22].

## 2 Results

### 2.1 Chemistry

**Figure 2:**
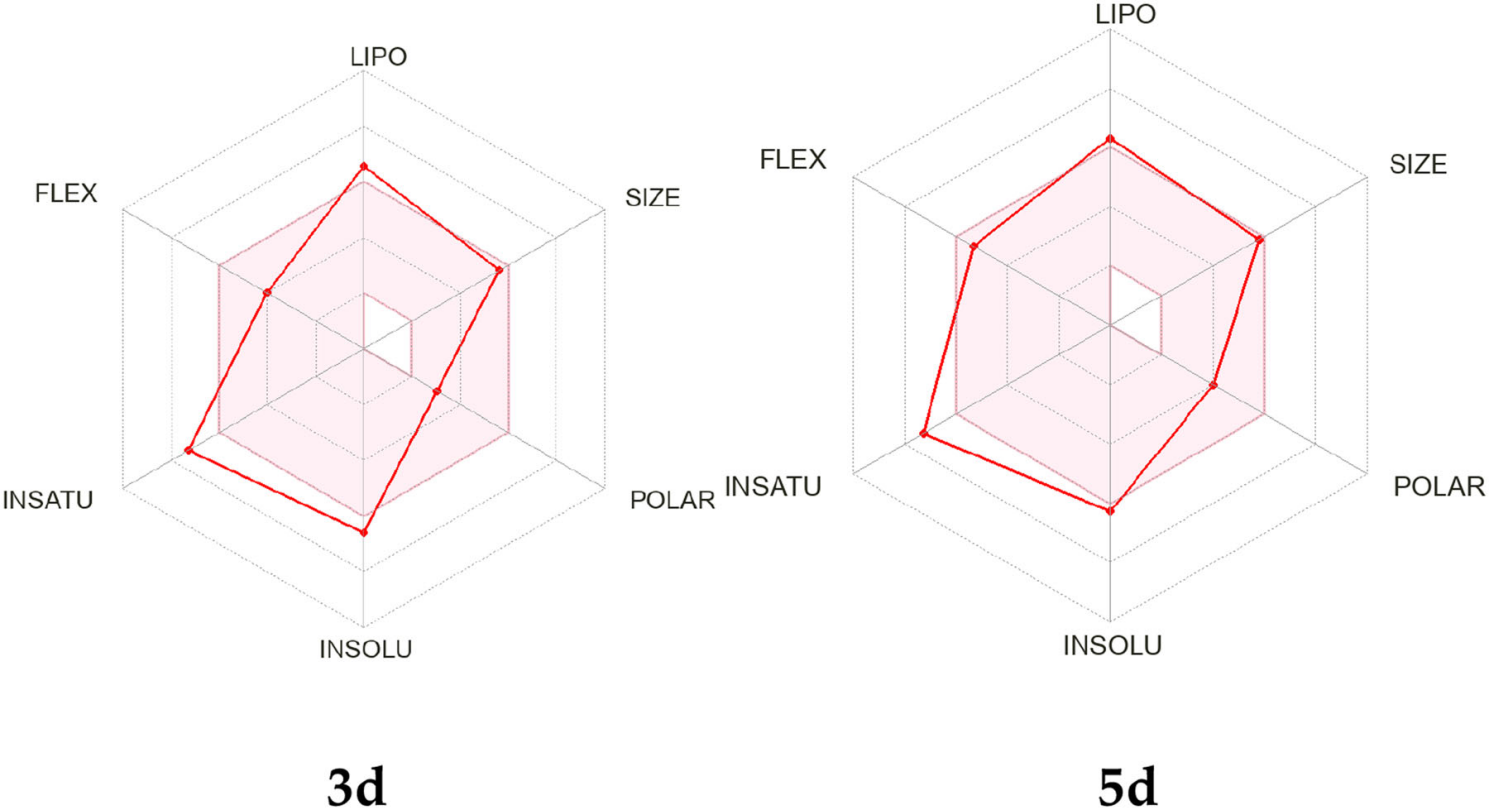
SwissADME bioavailability chart of the compounds **3d** and **5d** as representantives.

**Scheme 1:**
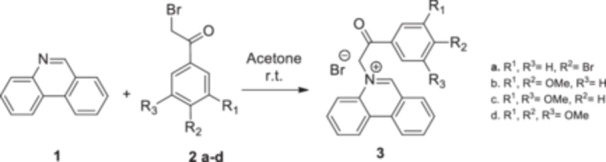
Reaction pathway to monoquaternary salts **3a–d**.

The strategy to build the desired series of target Phenstatin analogs (Figure 2) consisted of two main synthetic steps [23, 24], starting from the N‐heterocycle—phenanthridine to be fused to the pyrrrole ring via the monoquaternary phenanthridinium salts. Four derivatives were synthesized in each step, differing by the substituent at the phenyl ring. First, monoquaternary salts **3a–d**, were prepared by the direct reaction of phenanthridine, with 2‐bromo‐acetophenones **2** in acetone, at room temperature (r.t.) (Scheme 1).

**Scheme 2:**
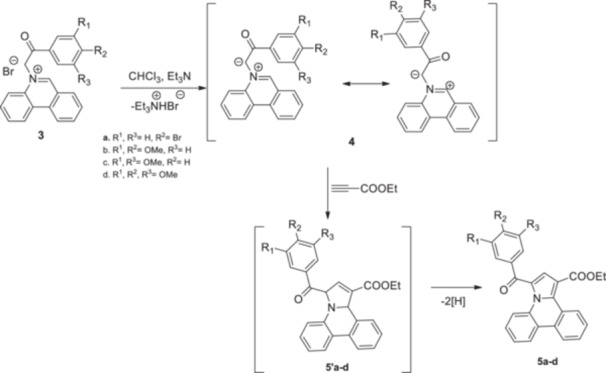
Reaction pathway to compounds **5a–d**.

The second step consisted of the in situ generation of the cycloimmonium ylides **4a–d** from the corresponding salts under triethylamine treatment. The in situ‐formed ylides acted as 1,3‐dipoles when reacted with ethyl propiolate, following a Huisgen [3 + 2] cycloaddition. Initially formed unstable intermediates **5′a–d** undergo an aromatization process under the current reaction conditions, leading to target compounds **5a–d** in 69%–77% yields (Scheme 2).

The structures of all synthesized compounds were confirmed through NMR spectroscopy, IR and MS analysis. In‐depth information about proton‐proton and proton‐carbon spin systems was obtained from homo‐ and heteronuclear bidimensional correlation experiments like ^1^H,^1^H‐COSY, ^1^H,^13^C‐HSQC and ^1^H,^13^C‐HMBC.

The monoquaternary salts formation was followed from the proton NMR spectra, by the downfield shift of the methylene signal around 7.0 ppm. Additionally, methylene‐nitrogen correlations were visible in the ^1^H,^15^N‐HMBC spectra, supporting the acetophenones N‐substitution. The successful synthesis of pyrrolo[1,2‐f]phenanthridines was evident from the proton spectra as well. Thus, the disappearance of the methylene (singlet at 7.0 ppm) and H‐6 (singlet at 10.0 ppm) signals, followed by the new signals associated with ethoxy residue (triplet at 1.35 ppm and quartet at 4.35 ppm) and H‐2 (singlet at 7.56 ppm) were correlated with the formation of the pyrrole cycle.

### 2.2 In Silico ADME and Toxicity Predictions

The results of the predicted parameters, including molecular properties, pharmacokinetics, drug‐likeness, and medicinal chemistry for compounds **3a–d** and **5a–d** were evaluated and the results are presented in Table 1.

**Table 1:** In silico prediction of ADME parameters for compounds 3a–d and 5a–d.

ADME parameter	3a	3b	3c	3d	5a	5b	5c	5d
*Physicochemical properties*								
Molecular weight	457.16 g/mol	438.31 g/mol	438.31 g/mol	468.34 g/mol	472.33 g/mol	453.49 g/mol	453.49 g/mol	483.51 g/mol
Log *P*_o/w_ (MLOGP)	4.42	3.06	3.06	2.71	4.54	3.27	3.27	2.93
Number of H‐bond acceptors	1	3	3	4	3	5	5	6
Number of rotatable bonds	3	5	5	6	5	7	7	8
TPSA	20.95 Å²	39.41 Å²	39.41 Å²	48.64 Å²	47.78 Å²	66.24 Å²	66.24 Å²	75.47 Å²
*Pharmacokinetics*								
Gastrointestinal (GI) absorption	High							
Blood‐brain barrier (BBB) permeant	Yes				No			
P‐gp substrate	Yes				No			
*Drug likeness*								
Log S (ESOL)	−7.31	−6.53	−6.53	−6.60	−6.94	−6.17	−6.17	−6.25
Water solubility class	Poorly soluble							
Lipinski rule	Yes; 1 violation: MLOGP > 4.15	Yes; 0 violation			Yes; 1 violation: MLOGP > 4.15	Yes; 0 violation		
Veber rule	Yes							
Bioavailability	0.55							
*Medicinal chemistry*								
PAINS alerts	1 alert: het_pyridiniums_A				0 alert			
Synthetic accessibility	2.05	2.48	2.48	2.72	2.93	3.30	3.30	3.50

The ADME profile of the compounds **3a–d** and **5a–d** offers critical insights into their pharmacokinetic properties and overall suitability for drug development.

Regarding the physicochemical properties, the molecular weights of these compounds range from 438.31 to 483.51 g/mol. These values are within the acceptable range for drug‐like molecules (typically ≤ 500 g/mol), though the higher molecular weights of **5a** and **5d** could potentially limit their absorption and bioavailability. In terms of lipophilicity, the Log Po/w (MLOGP) values span from 2.71 to 4.54, with **5a** and **3a** being more lipophilic. This suggests that these compounds may exhibit good membrane permeability but could face challenges with solubility. On the other hand, **3d** and **5d**, having lower Log Po/w values, are more hydrophilic and may be better soluble, although their permeability could be compromised. The number of hydrogen bond acceptors ranges from 3 to 6, influencing the solubility and permeability. Compounds like **3a** and **3b**, with fewer HBAs, may have better permeability, while compounds such as **5d** and **3d** could encounter issues with membrane passage due to an increased number of hydrogen bonding interactions.

The number of rotatable bonds, which ranges from 3 to 8, impacts the flexibility of these compounds. More flexible molecules, such as **3d**, and **5d**, may exhibit broader interactions with their targets but might suffer from reduced specificity. Meanwhile, **3a**, with fewer rotatable bonds, is more rigid, which could enhance target specificity. Similarly, the Topological Polar Surface Area (TPSA) values vary from 20.95 Å² to 75.47 Å². Compounds with lower TPSA values, such as **3a** and **3b**, are likely to have better membrane permeability, whereas those with higher TPSA, such as **5c** and **5d**, may face challenges crossing biological membranes due to their increased polarity.

In terms of pharmacokinetics, all compounds are predicted to have high gastrointestinal absorption, which suggests that they should be efficiently absorbed in the gastrointestinal tract. However, the ability to cross the blood‐brain barrier (BBB) varies. Compounds **3a–d** are predicted to be BBB permeant, indicating their potential utility for treating central nervous system (CNS) disorders. In contrast, compounds **5a–d** are not expected to cross the BBB, limiting its applicability in CNS conditions. The P‐glycoprotein (P‐gp) substrate status is predicted for **3a–d**, which could hinder their bioavailability due to P‐gp‐mediated efflux. Compounds that do not interact with P‐gp, such as **5a–d**, are likely to have improved bioavailability.

The drug‐likeness of these compounds is also assessed, with Log S values indicating poor solubility across the board, ranging from −6.17 to −7.31. This poor solubility could limit their oral bioavailability and suggests that formulation strategies or structural modifications might be required to enhance solubility. All the compounds pass Lipinski's Rule of 5, with most violating at most one rule, typically related to MLOGP > 4.15, which is indicative of higher lipophilicity in **3a** and **5a**. Additionally, all compounds pass the Veber rule, suggesting they could exhibit favorable oral bioavailability. The bioavailability of these compounds is predicted to be moderate, with values around 0.55, indicating that while they may be absorbed, significant first‐pass metabolism could reduce their overall bioavailability.

From a medicinal chemistry perspective, compounds **3a–d** have a PAINS alert related to a pyridinium derivative, suggesting potential issues in screening assays. The rest of the compounds show no such alerts, making them more promising for further development. Synthetic accessibility values indicate that compounds **3a** and **3b** are easier to synthesize, with lower accessibility scores, while **5c** and **5 d** are more complex to produce, which might impact their scalability in pharmaceutical production.

The chart generated from the SwissADME QSAR web tool, regarding the accessibility of compounds **3d** and **5d** (chosen as representantives) to be orally bioavailable, is presented in Figure 2.

This radar involves six parameters: lipophilicity (LIPO), size, polarity (POLAR), insolubility (INSOLU), unsaturation (INSATU), and flexibility (FLEX) of the tested compound, and is represented by a red line integrated into a pink area. Molecules that fall within the pink region of the radar are considered drug‐like.

Compounds **3a–d** exhibited compliance to three of six rules violating the ratio of hybridized sp [3] atoms to the total number of C atoms, lipophilicity and solubility. Meanwhile, compounds **5a–d** also showed partial compliance, but with a better overall profile than compounds **3a–d**, except for the INSATU parameter, which was similarly deviated. Notably, the profiles of compounds **3a–d** and **5a–d** are highly similar within each series, despite the differences in phenyl substitution. Taken together, these predicted data show a promising ADME and drug‐likeness profile for proposed compounds.

The predicted toxicity spectrum is represented by a list of activities with probabilities “to be active” (P_a_) and “to be inactive” (P_i_). The obtained results, presented in Table 2, show predicted cytotoxicity (Pa > Pi and Pa > 0.3) against several cancer cell lines (Table 2). The fact that no normal human cell lines appeared on the list could be an indication of a good selectivity of tested compounds against cancer cell lines.

**Table 2:** Results of the prediction for cytotoxicity of compounds 5a–d.

Pa	Pi	Cell‐line	Description	Tissue/organ	Type	IAP
**5a**						
0.635	0.002	SNU‐398	Hepatocellular carcinoma	Liver	Carcinoma	0.970
0.386	0.011	RPMI‐7951	Malignant Melanoma	Skin	Melanoma	0.854
0.371	0.158	MCF7	Breast carcinoma	Breast	Carcinoma	0.836
0.336	0.036	CL1‐0	Lung adenocarcinoma	Lung	Adenocarcinoma	0.830
0.334	0.049	CCRF‐CEM	Childhood T acute lymphoblastic leukemia	Blood	Leukemia	0.913
0.329	0.057	SNB‐75	Glioblastoma	Nervous system	Glioblastoma	0.877
0.322	0.023	C6	Glioma	Brain	Glioma	0.980
0.319	0.065	NCI‐H522	Non‐small cell lung carcinoma	Lung	Carcinoma	0.877
0.310	0.022	A‐375	Malignant melanoma	Skin	Melanoma	0.931
0.305	0.286	A2780cisR	Cisplatin‐resistant ovarian carcinoma	Ovarium	Carcinoma	0.838
**5c**						
0.631	0.002	SNU‐398	Hepatocellular carcinoma	Liver	Carcinoma	0.970
0.444	0.026	SNB‐75	Glioblastoma	Nervous system	Glioblastoma	0.877
0.440	0.028	NCI‐H522	Non‐small cell lung carcinoma	Lung	Carcinoma	0.877
0.421	0.008	CL1‐0	Lung adenocarcinoma	Lung	Adenocarcinoma	0.830
0.411	0.006	RPMI‐7951	Malignant Melanoma	Skin	Melanoma	0.854
0.392	0.034	CCRF‐CEM	Childhood T acute lymphoblastic leukemia	Blood	Leukemia	0.913
0.387	0.036	M14	Melanoma	Skin	Melanoma	0.894
0.376	0.046	KM12	Colon adenocarcinoma	Colon	Adenocarcinoma	0.875
0.366	0.059	HCT‐116	Colon carcinoma	Colon	Carcinoma	0.890
0.354	0.104	SNU‐475	Hepatocellular carcinoma	Liver	Carcinoma	0.819
0.354	0.170	MCF7	Breast carcinoma	Breast	Carcinoma	0.836
0.351	0.107	CA46	Burkitts Lymphoma	Blood	Lymphoma	0.810
0.344	0.016	A‐375	Malignant melanoma	Skin	Melanoma	0.931
0.329	0.022	C6	Glioma	Brain	Glioma	0.980
**5b**						
0.624	0.002	SNU‐398	Hepatocellular carcinoma	Liver	Carcinoma	0.970
0.453	0.025	SNB‐75	Glioblastoma	Nervous system	Glioblastoma	0.877
0.442	0.027	NCI‐H522	Non‐small cell lung carcinoma	Lung	Carcinoma	0.877
0.415	0.006	RPMI‐7951	Malignant Melanoma	Skin	Melanoma	0.854
0.412	0.010	CL1‐0	Lung adenocarcinoma	Lung	Adenocarcinoma	0.830
0.402	0.033	M14	Melanoma	Skin	Melanoma	0.894
0.395	0.033	CCRF‐CEM	Childhood T acute lymphoblastic leukemia	Blood	Leukemia	0.913
0.382	0.044	KM12	Colon adenocarcinoma	Colon	Adenocarcinoma	0.875
0.367	0.162	MCF7	Breast carcinoma	Breast	Carcinoma	0.836
0.356	0.098	CA46	Burkitts Lymphoma	Blood	Lymphoma	0.810
0.352	0.015	A‐375	Malignant melanoma	Skin	Melanoma	0.931
**5d**						
0.631	0.002	SNU‐398	Hepatocellular carcinoma	Liver	Carcinoma	0.970
0.489	0.021	SNB‐75	Glioblastoma	Nervous system	Glioblastoma	0.877
0.466	0.023	NCI‐H522	Non‐small cell lung carcinoma	Lung	Carcinoma	0.877
0.447	0.023	M14	Melanoma	Skin	Melanoma	0.894
0.426	0.005	RPMI‐7951	Malignant Melanoma	Skin	Melanoma	0.854
0.418	0.029	CCRF‐CEM	Childhood T acute lymphoblastic leukemia	Blood	Leukemia	0.913
0.412	0.133	MCF7	Breast carcinoma	Breast	Carcinoma	0.836
0.407	0.037	KM12	Colon adenocarcinoma	Colon	Adenocarcinoma	0.875
0.400	0.056	HT‐29	Colon adenocarcinoma	Colon	Adenocarcinoma	0.888
0.392	0.014	CL1‐0	Lung adenocarcinoma	Lung	Adenocarcinoma	0.830
0.386	0.011	A‐375	Malignant melanoma	Skin	Melanoma	0.931
0.365	0.080	CA46	Burkitts Lymphoma	Blood	Lymphoma	0.810
0.353	0.063	HCT‐116	Colon carcinoma	Colon	Carcinoma	0.890
0.335	0.068	DU‐145	Prostate carcinoma	Prostate	Carcinoma	0.896
0.333	0.048	A498	Renal carcinoma	Kidney	Carcinoma	0.892
0.330	0.012	MDA‐ MB‐453	Breast adenocarcinoma	Breast	Adenocarcinoma	0.888
0.329	0.022	C6	Glioma	Brain	Glioma	0.980
0.329	0.055	MES‐SA	Uterine corpus sarcoma	Uterus	Sarcoma	0.815
0.320	0.055	SK‐MEL‐5	Melanoma	Skin	Melanoma	0.903

### 2.3 Molecular Modeling

Molecular docking studies revealed that all synthesized pyrrolo‐fused derivatives display improved affinity at the colchicine binding site compared to Colchicine (−8.69 kcal/mol) and Phenstatin (−7.66 kcal/mol). The most promising ligands belong to series **5**, with compounds **5d** and **5c** showing the lowest binding energies of −11.73 and −11.65 kcal/mol, respectively, indicating strong and favorable interactions within the binding pocket. Compound **5d**, being the ligand with the best binding affinity among all studied compounds, is also the only one to form a hydrogen bond with a residue from α‐chain, namely αASN‐101, while also maintaining interaction with βASN‐258—a distinctive feature that likely contributes to its enhanced stabilization. Compounds **5b** and **5c** each form two hydrogen bonds with βASN‐258 and βLYS‐254, residues commonly involved in ligand stabilization at the binding site. Compound **5a**, however, displays a weaker interaction profile, forming only one hydrogen bond with βASN‐258.

Compounds from series **3** display lower binding affinities overall, with **3a** being the most active (−10.78 kcal/mol). Notably, only two docking clusters were identified for **3a**, implying a very high specificity for the binding site and this is a unique phenomenon compared to the other binding results. In terms of interactions, **3a** forms two halogen bonds with βVAL‐315 and βASN‐350, as well as a hydrogen bond with βASN‐258, which together contribute to the stability of the ligand‐protein complex. In contrast, compound **3d** forms only one hydrogen bond with βASN‐258, indicating a weaker interaction and lower affinity. Compounds **3b** and **3c** exhibit a similar interaction profile, each forming two hydrogen bonds with residues βLYS‐254 and βASN‐258, further supporting the role of these residues in ligand anchoring.

As regards the reference compounds, their behaviors are contrasting. **Colchicine**, although known for its biological activity, does not form any hydrogen bonds within the complex, suggesting a binding mechanism that may be dominated by hydrophobic interactions. On the other hand, **Phenstatin** forms five hydrogen bonds, involving βTHR‐179, two with βGLN‐247, as well as βLYS‐352 and βGLN‐336, highlighting its polar nature and potential for extensive stabilization in the active site.

βASN‐258 forms a hydrogen bond with a C=O group of ligands by donating a hydrogen atom from the NH₂ of its side chain amide group to the carbonyl oxygen, which acts as acceptor. The recurring involvement of this residue across almost all ligands underscores its critical role in ligand recognition and complex stabilization. Overall, the synthesized pyrrolo‐fused derivatives demonstrate considerable potential as colchicine‐site binders, with compounds **5d** and **5c** exhibiting promising binding profiles and promising interaction networks compared to the reference ligands.

The 2D interaction diagrams display the interactions between ligands and our protein of interest, with a cutoff distance of 4 Å, enough to capture the predominant electrostatic interactions and hydrogen bonding interactions which are essential in protein‐ligand complexes. The line around the ligands indicates the nature of its interactions with the surrounding amino acid residues within the binding pocket. The pink lines and arrows represent hydrogen bonds; the arrow indicates the donated hydrogen bond. Protein residues are represented as a guitar pick linked together by strings. The orientation of each guitar pick conveys important structural information: when the picks are pointing away from the ligand, it is suggested that the backbone of that residue is pointing toward the ligand. When the guitar pick is pointing towards the ligand, this indicates that the side chain of that residue is facing the ligand. Finally, the gray shaded areas are accessible to the solvent or other molecules (Table 3).

**Table 3:** The binding modes of the most promising ligand conformations were compared to those of the reference compounds Colchicine and Phenstatin within the known colchicine‐binding pocket of tubulin.

Ligand	Lowest binding energy (kcal/mol)	Binding conformation	2D interaction diagram
**Colchicine**	−8.69	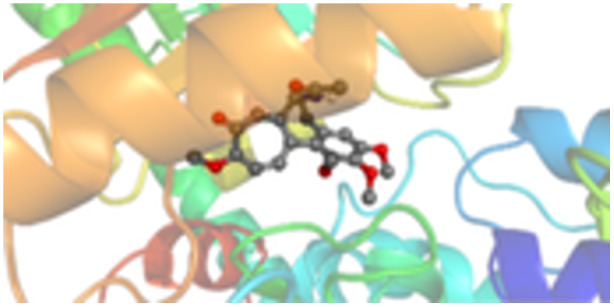	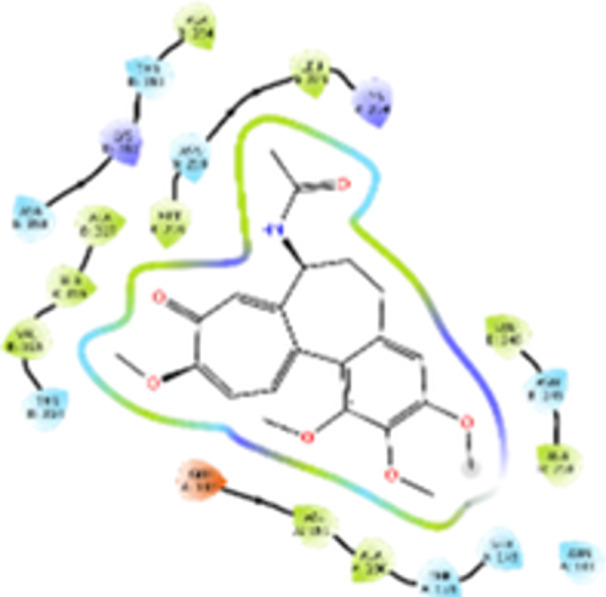
**Phenstatin**	−7.66	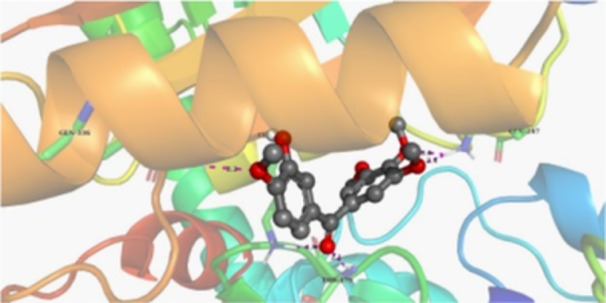	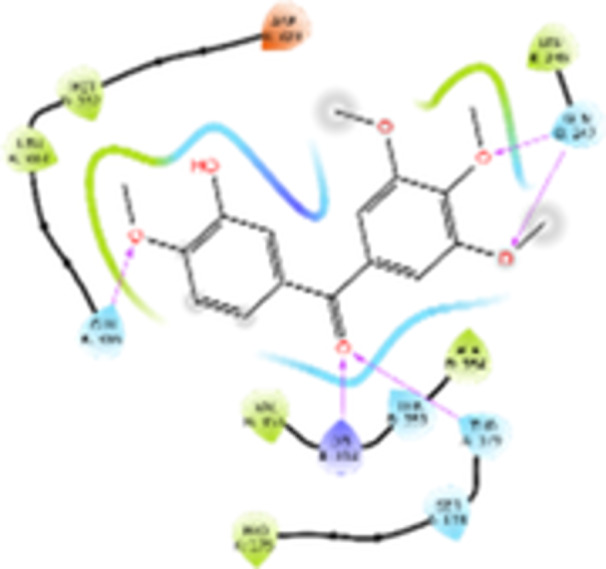
**3a**	−10.74	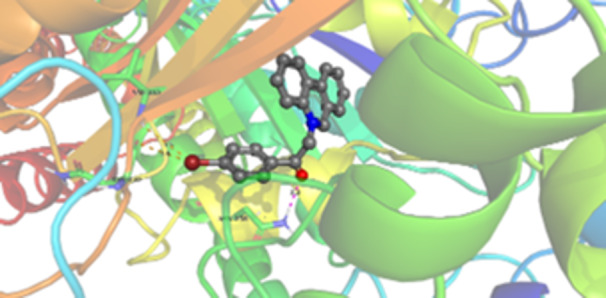	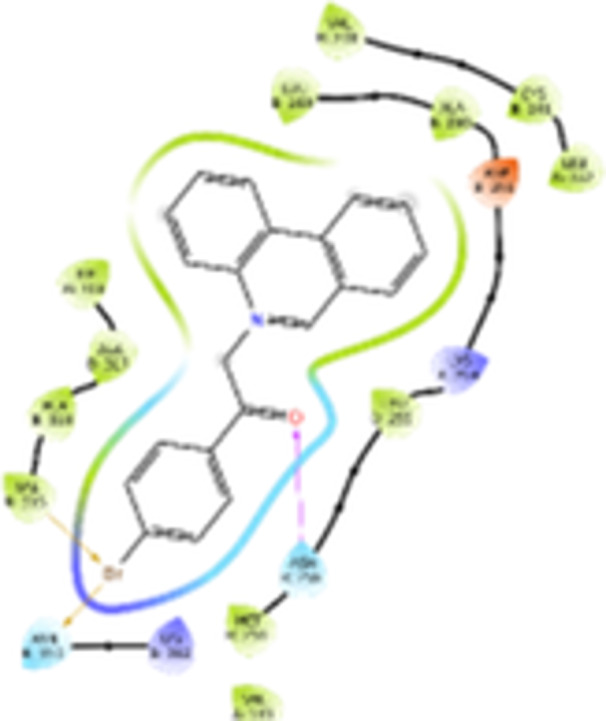
**3b**	−9.90	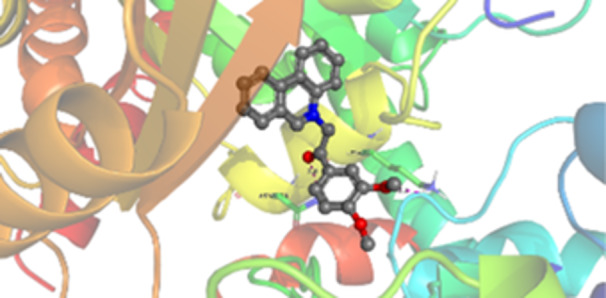	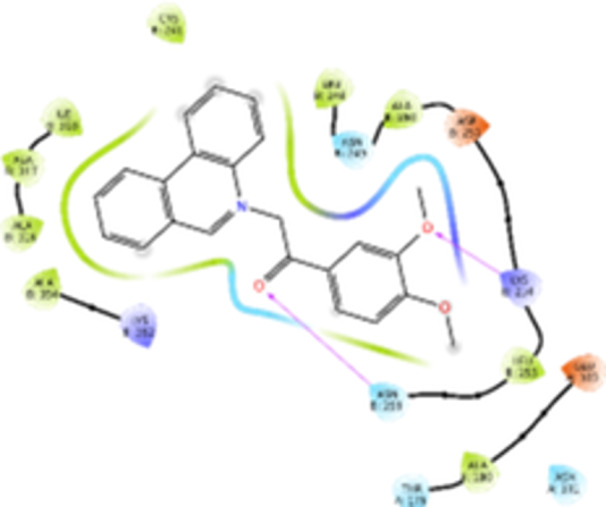
**3c**	−9.84	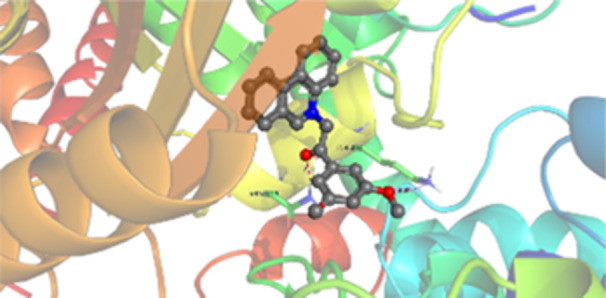	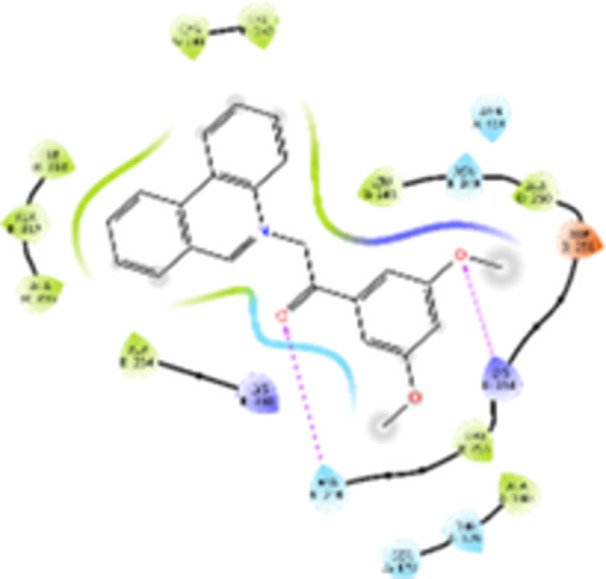
**3d**	−10.03	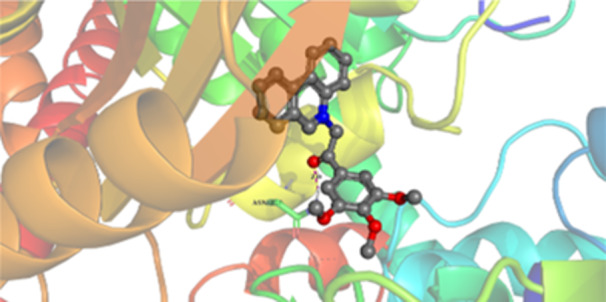	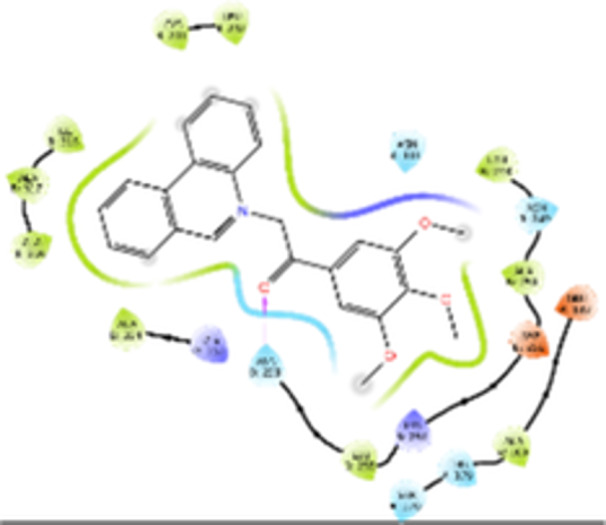
**5a**	−11.53	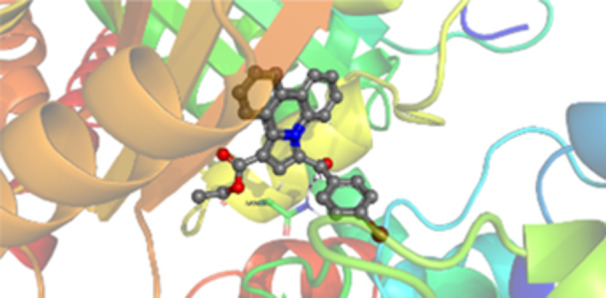	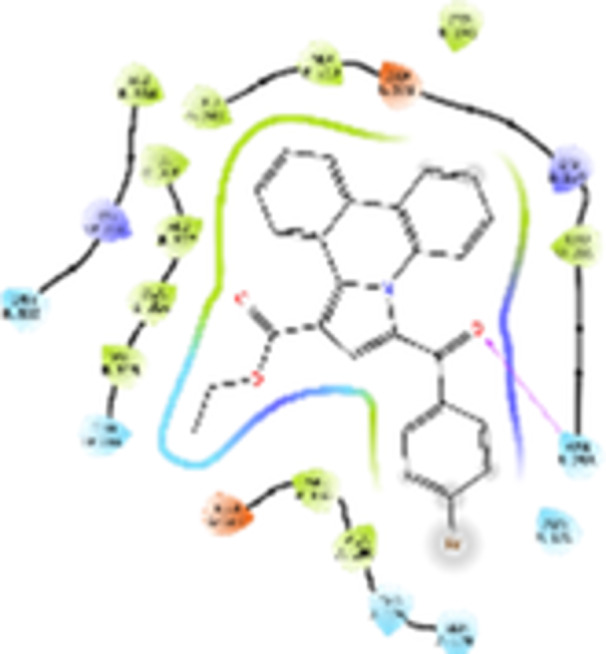
**5b**	−11.58	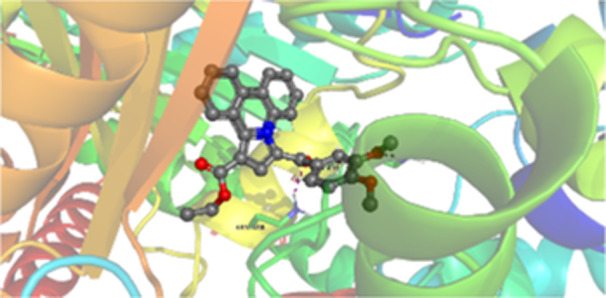	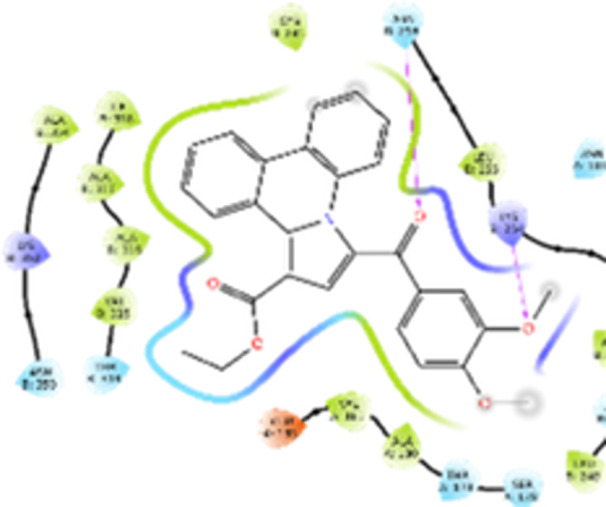
**5c**	−11.65	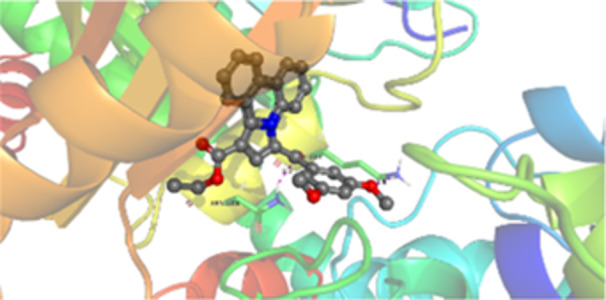	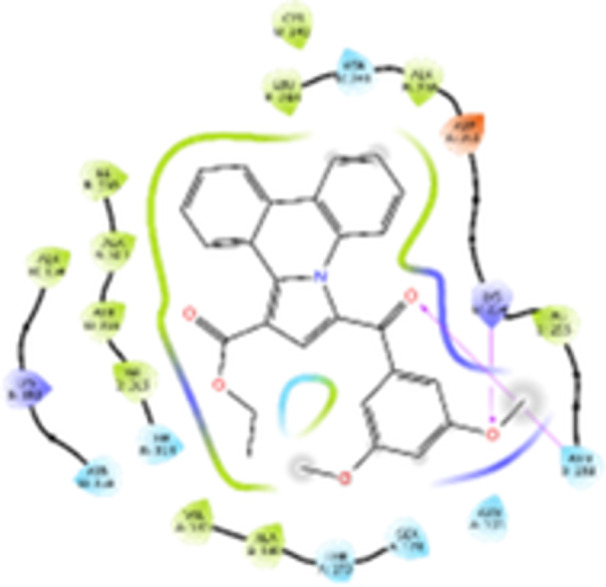
**5d**	−11.73	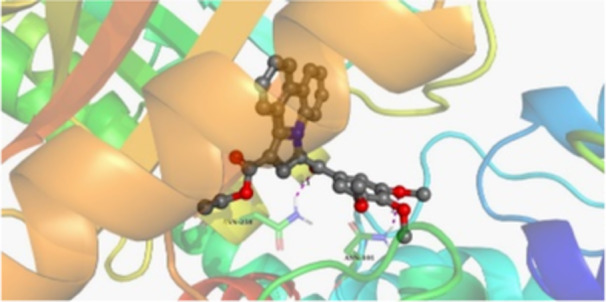	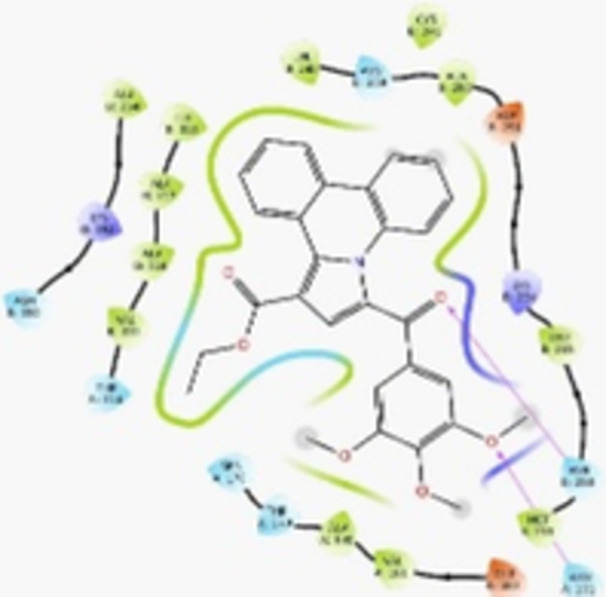

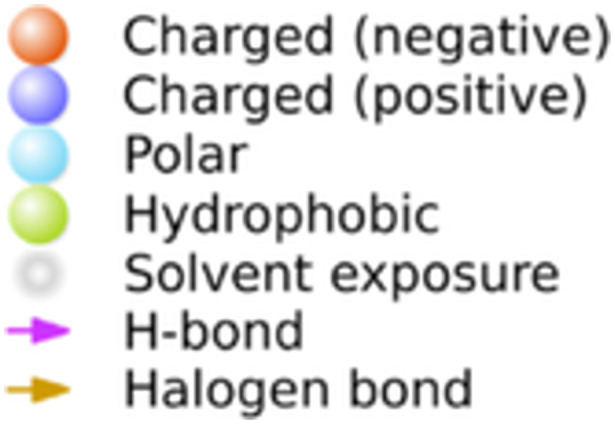

All ligands, including the references, are depicted using the CPK color scheme. Hydrogen bonds are represented as magenta dotted lines, while halogen bonds are depicted in orange.

**Figure 3:**
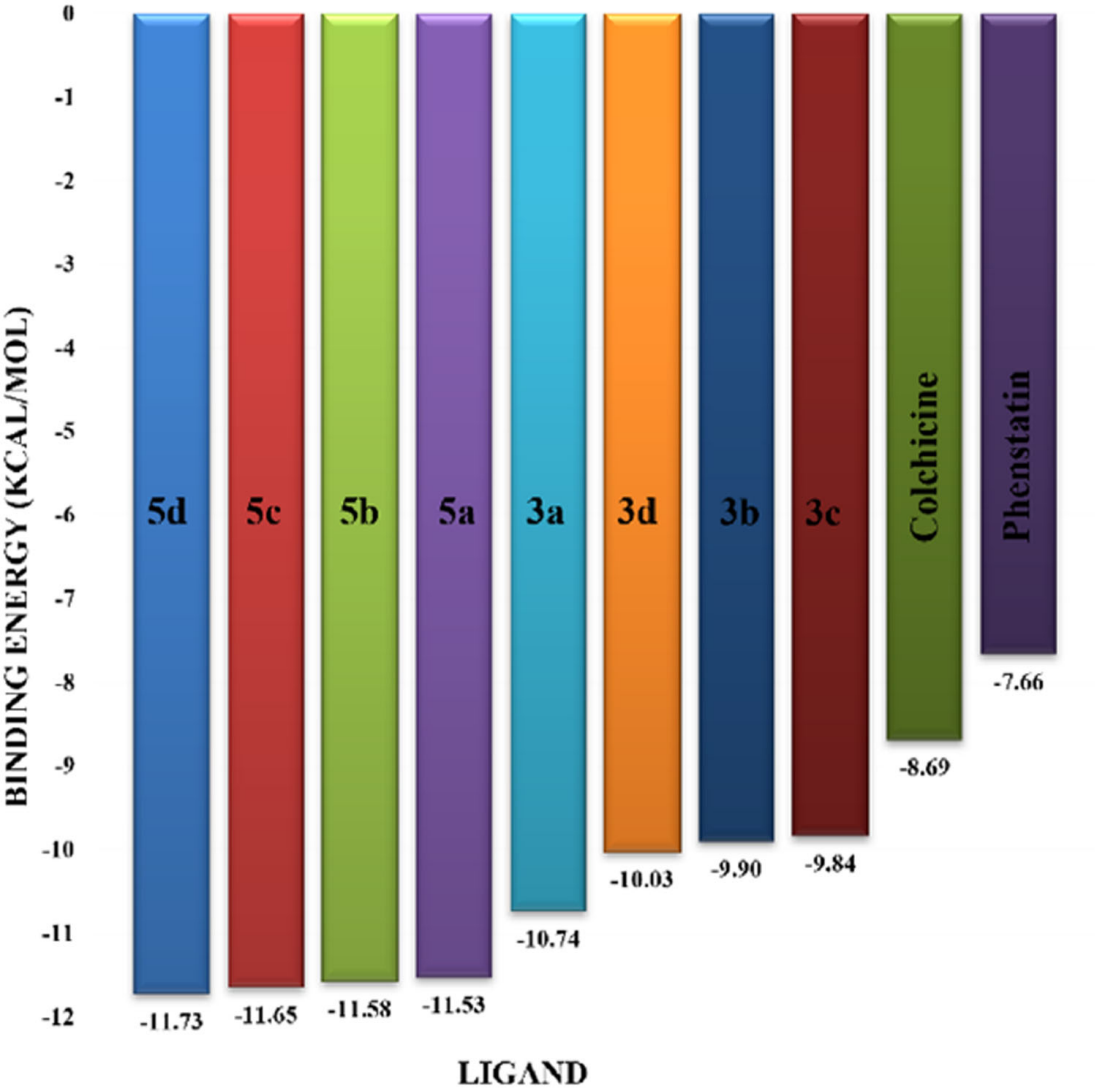
Binding energies of ligands docked to tubulin.

Figure 3 presents the ligand efficiency hierarchy, determined using the energy data from Table 3.

### 2.4 Cytotoxicity Evaluation

**Figure 4:**
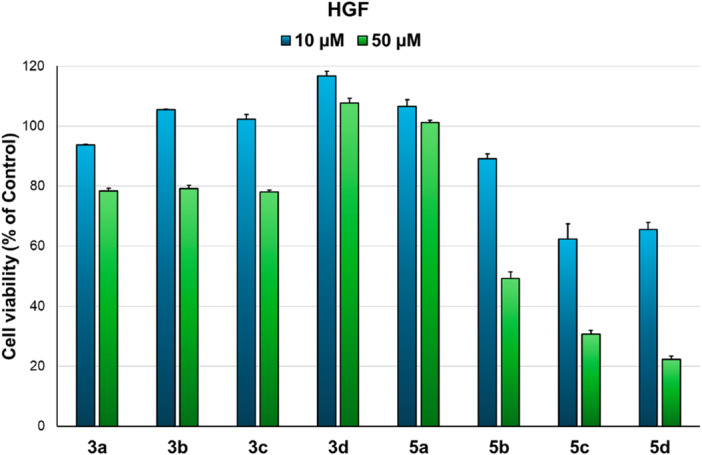
Cell viability of normal fibroblasts (HGF) incubated with compounds (10 and 50 µM) for 24 h.

The evaluation of anticancer potential, expressed as cytotoxicity, was performed in two steps: first on normal fibroblasts (HGF) and second on cancer cell lines. First, cytotoxicity tests performed on HGF cells (Figure 4) revealed that, at the tested concentrations (10 and 50 µM), monoquaternary salts (**3a–d**) are biocompatible (cell viability > 78%). Interestingly, compound **3d** induced a slight proliferation of HGF cells at 10 µM concentration (+17%). However, we found that, from the fused pyrrol‐phenanthridine compounds, only compound **5a** is biocompatible at both tested concentrations and derivative **5b** only at 10 µM concentration, determining cell viability above 89%. Compounds **5c** and **5d** induced a slight cytotoxic effect in normal fibroblasts at 10 µM concentration (62% and 65% cell viability, respectively) and a more pronounced effect at 50 µM (31% and 22% cell viability, respectively). Therefore, for the next step in anticancer evaluation we chose to test only compounds **3a–d**, **5a**, and **5b** at the selected concentrations.

**Figure 5:**
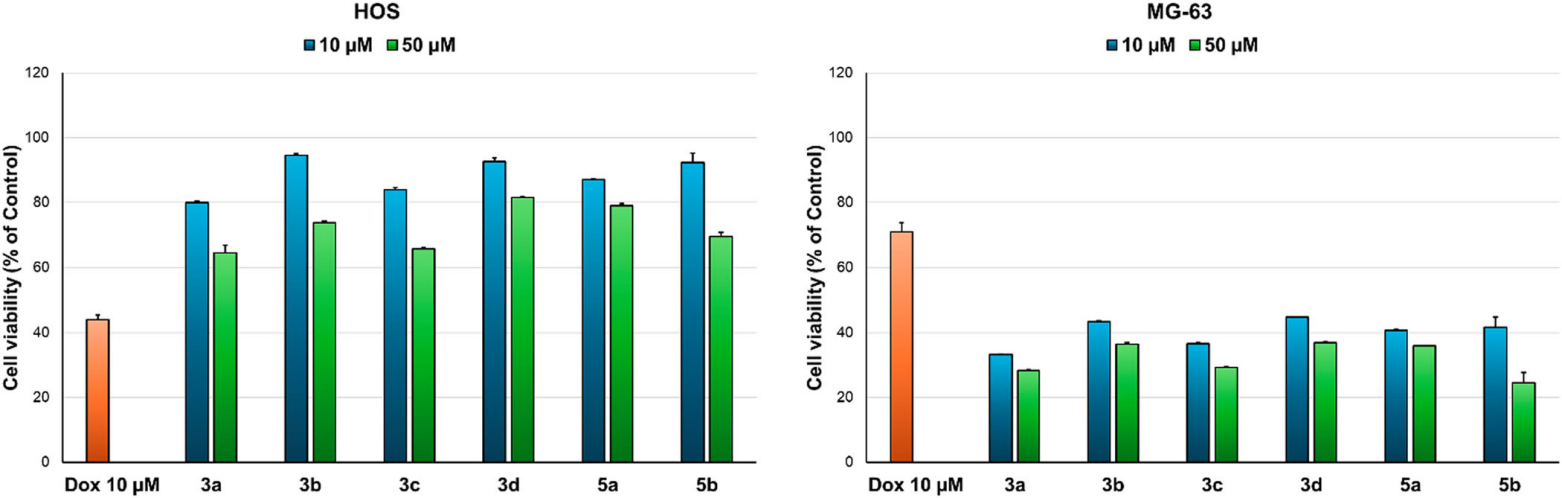
Cytotoxicity of selected compounds (**3a–d**, **5a**, and **5b**) at 10 and 50 µM on human osteosarcoma cell lines (HOS and MG‐63) after 24 h incubation.

Osteosarcoma is one of the most common types of bone cancer, characterized by poor prognosis and genetic abnormalities and variability [25, 26]. Therapeutic regimens implemented in the 1980s have increased survival rates, but further advances have stalled despite ongoing efforts to develop novel approaches for osteosarcoma treatment [26]. Furthermore, osteosarcoma often presents with early pulmonary metastasis, which decreases survival time and therapeutic efficacy [25]. Park and colleagues showed that sanguinarine induced apoptosis in MG‐63 cells at very low concentrations in a time‐dependent manner, more effectively than in SaOS‐2 cells [27], thus, in this study, selected compounds (**3a–d**, **5a**, and **5b**) were evaluated on two osteosarcoma cell lines (HOS and MG‐63) and the results (Figure 5) showed that all tested compounds present very good activity at the tested concentrations on the MG‐63 line, reducing cell viability by more than 55% at 10 µM. However, the cytotoxic effects of the compounds on the HOS cell line were poor (cell viability = 64%–81% at 50 µM). This could be explained by the molecular heterogeneity of osteosarcoma subtypes [25], and, implicitly, between these two cell lines. MG‐63 cell line was derived from the bone tumor of a Caucasian male (14 years old) and has fibroblast morphology and rapid proliferation [28], while HOS cell line was isolated from a Caucasian female (13 years old) and has a mixed fibroblast and epithelial morphology [29]. It has been demonstrated that HOS and MG‐63 cell lines present some phenotypic and genetic differences, but their metastatic behavior is similar [30].

**Figure 6:**
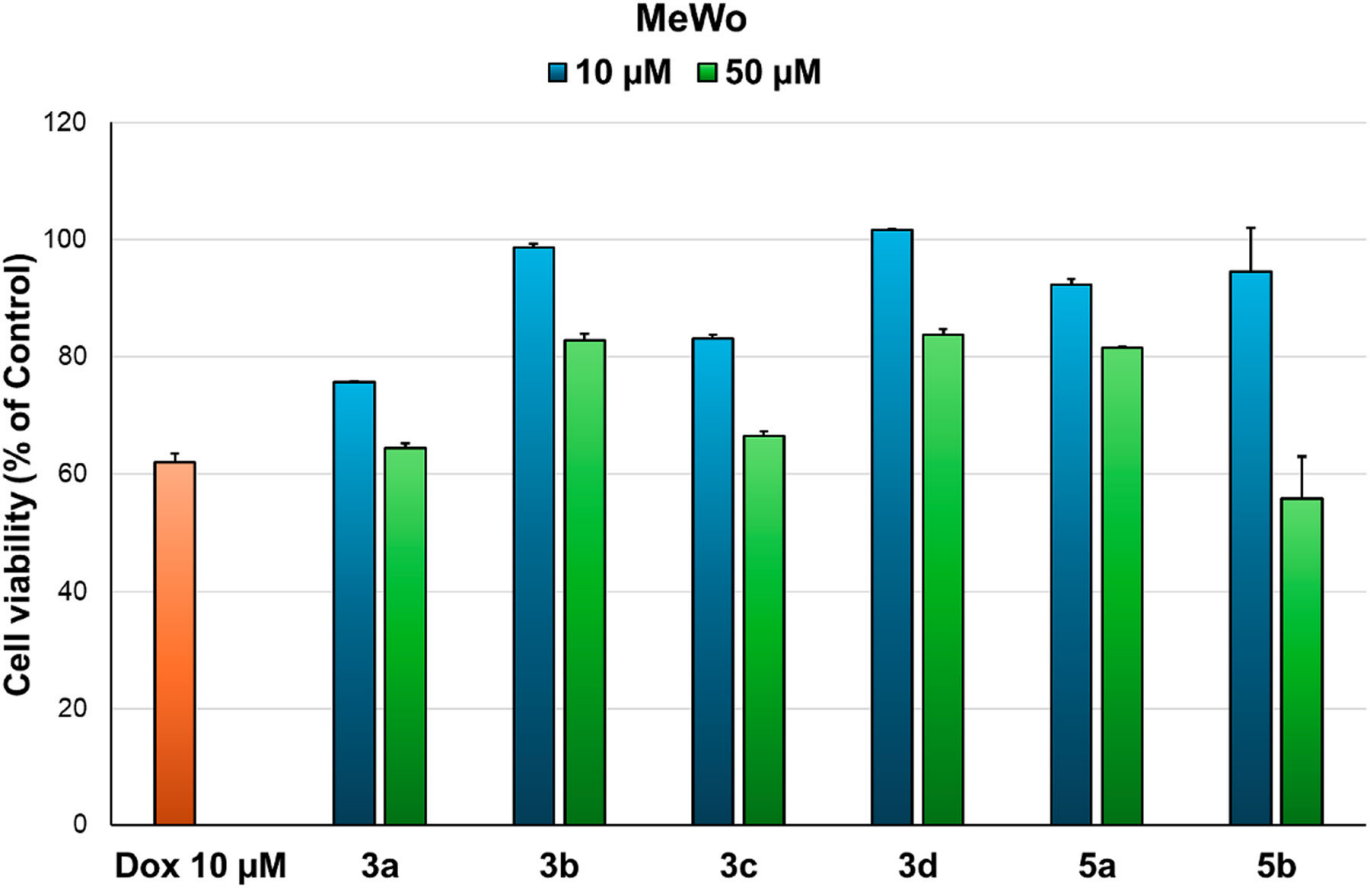
Cytotoxicity of selected compounds (**3a–d**, **5a**, and **5b**) at 10 and 50 µM on malignant melanoma cell line (MeWo) after 24 h incubation.

Given the *in silico* CLC in silico prediction results obtained, which show that **5a** and **5b** should be effective on melanoma (2 cell lines each in top 10 predicted), the selected compounds were also evaluated on MeWo melanoma cell line. The results showed that, while compounds **3a** and **3c** showed borderline cytotoxicity on MeWo cells at 50 µM, compound **5b** presented the best activity of the selected compounds at this concentration (Figure 6). However, compound **5b** was cytotoxic at 50 µM on HGF cells, indicating that it is not selective for melanoma. The discrepancy between the theoretical and experimental results could be explained by the intrinsic differences between cancer cell lines derived from patients, such as age of patient, genetic and phenotypic features [31] M14, RPMI‐7951, and A‐375 cell lines have an epithelial morphology and are derived from lesions of younger patients (male 33 years old, female 18 years old and female 54 years old, respectively), while MeWo cells are fibroblast‐like and derived from an older male patient (male 78 years old).

Based on these findings, compounds **3a–d**, **5a**, and **5b** have good potential for the treatment of some osteosarcoma subtypes at low concentrations, but further in‐depth studies are needed to establish the mechanism of action and the safety profile of these compounds. Furthermore, while CLC in silico predictions indicated the potential of compounds **5a** and **5b** to induce cytotoxic effects in melanoma cells, but not in osteosarcoma cells, we observed that, experimentally, their activity is reversed, being effective on MG‐63 osteosarcoma cell line, but not on MeWo melanoma cells. This shows that, while CLC in silico predictions are a useful tool in determining potential cytotoxic activity of synthetic compounds and guiding experimental procedures, they can also be misleading sometimes, probably due to intrinsic metabolic and constitutive differences between cell lines and cell types.

## 3 Conclusions

In this manuscript, we report the successful synthesis of four novel monoquaternary salts and four fused pyrrol‐phenanthridine compounds. All compounds were fully characterized and confirmed through NMR, FT‐IR, and MS analysis. Based on theoretical predictions, we evaluated their cytotoxic activity alongside biocompatibility assessments.

The four salts and two cycloadducts were tested on osteosarcoma cell lines HOS and MG‐63. While no significant differences were observed among the compounds, a clear distinction emerged between the cell lines. All compounds inhibited the MG‐63 cell line by approximately 50%, whereas inhibition of the HOS cell line ranged from 20% to 30%.

When tested on the MeWo melanoma cell line, despite promising theoretical predictions, the compounds showed no significant activity.

Compounds **3a–d**, **5a**, and **5b** demonstrated good biocompatibility at 10 and 50 µM, along with good cytotoxic effects against the MG‐63 osteosarcoma cell line, highlighting their therapeutic potential in osteosarcoma treatment. The synthesized pyrrolo‐fused derivatives demonstrated considerable potential as colchicine‐site binders in molecular docking investigations, with compounds **5a** and **5d** exhibiting promising binding profiles and superior interaction networks compared to the reference ligands. These results demonstrate the potential of these compounds as anticancer candidates and support future additional research.

## 4 Materials and Methods

### 4.1 Chemistry

All of the commercially available reagents and solvents employed were used without further purification. The melting points were recorded on an A. Krüss Optronic Melting Point Meter KSPI and are uncorrected. Analytical thin‐layer chromatography was performed with commercial silica gel plates 60 F254 (Merck Darmstadt, Germany) and visualized with UV light (λmax = 254 or 365 nm).

The proton and carbon NMR spectra were recorded on Bruker Avance III 500 MHz and Bruker Avance NEO 400 MHz spectrometers, both equipped with 5 mm z‐gradient probes. The proton‐nitrogen HMBC experiments were recorded on Bruker Avance NEO 600 MHz spectrometer, equipped with an 5 mm, inverse detection, z‐gradient probe. Chemical shifts were reported in delta (δ) units, part per million (ppm) and coupling constants (J) in Hz. The following abbreviations were used to designate chemical shift multiplicities: s = singlet, d = doublet, t = triplet, q = quartet, m = multiplet.

IR spectra were recorded on a Shimadzu IRTracer‐100 instrument (Shimadzu U.S.A. Manufacturing Inc., Canby, OR, USA).

Mass spectra were acquired using a MALDI‐TOF/TOF Mass Spectrometer (ultrafleXtreme TM, Bruker Daltonics) equipped with a pulsed nitrogen UV laser (λmax 337 nm). The sample preparation procedure was the same as reported by [32]. Briefly, the mixture of MALDI matrix (α‐cyano‐4‐hydroxycinnamic acid) and sample solution in 2:1 ratio was deposited on a 384‐spot MALDI target plate (1 µL for each spot) using the dried‐droplet method. The loaded plate was then dried at room temperature before MALDI‐MS analysis. FlexControl 3.4 was used to optimize and acquire data using the following parameters: positive reflector mode, 20 kV acceleration voltage and acquisition mass range m/z 100–1600 Da. The laser power was set at 50%–70% of the maximum, and a total of 500 shots per acquisition were accumulated to obtain the final mass spectrum. The obtained spectra were processed using Bruker's FlexAnalysis 3.4 software. External calibration was performed using a peptide mixture standard solution from Bruker Daltonics, Bremen‐Germany.

#### 4.1.1 General Procedure for the Synthesis of Monoquaternary Salts **3a–d**

One millimole of phenanthridine was suspended in minimum volume of acetone (aprox 4–5 mL). Then, 1.1 mmol of reactive halide (2‐bromo‐1‐(4‐bromophenyl) ethanone **2a,** 2‐bromo‐1‐(3,4‐dimethoxyphenyl) ethanone **2b,** 2‐bromo‐1‐(3,5‐dimethoxyphenyl) ethanone **2c** or 2‐bromo‐1‐(3,4,5‐trimethoxyphenyl)ethanone **2d**) was added and the resulting mixture was stirred overnight at room temperature. The formed precipitate was filtered and washed with diethyl ether to give the desired product which was used in the next reaction without any further purification.

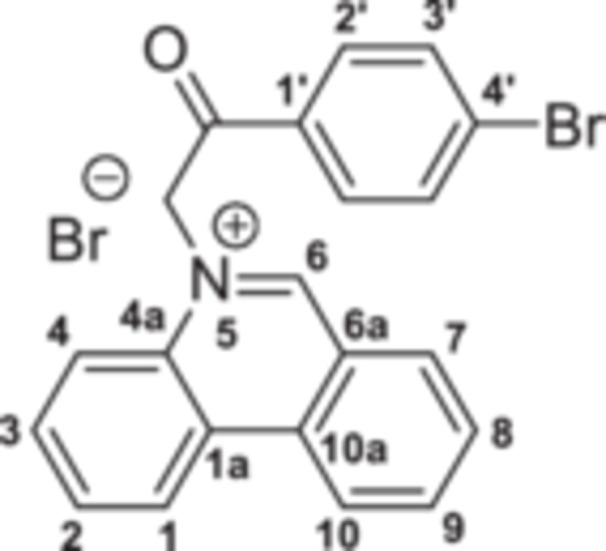

**5‐(2‐(4‐Bromophenyl)‐2‐oxoethyl)phenanthridin‐5‐ium bromide 3a:** Yellow solid; η = 97%, mp = 214°C–215°C; IR(ATR) ν(cm^−1^): 3249, 3197, 3053, 3005, 2906, 2883, 1770, 1739, 1712, 1681, 1626, 1585, 1537, 1448, 1398, 1338, 1242, 1223, 1068, 1014, 982, 818, 785, 756, 721, 638, 582, 525, 486.

^1^H‐NMR (DMSO‐d_6_, 500.1 MHz, δ (ppm)): 7.09 (2H, s, CH_2_), 7.96 (2H, d, ^*3*^ *J* = 8 Hz, H‐3’), 8.10 (1H, t, ^*3*^ *J* = 8 Hz, H‐3), 8.13 (2H, d, ^*3*^ *J* = 8 Hz, H‐2ʹ and H‐2), 8.17 (1H, t, ^*3*^ *J* = 8 Hz, H‐8), 8.50 (1H, t, ^*3*^ *J* = 8 Hz, H‐9), 8.51 (1H, d, ^*3*^ *J* = 8 Hz, H‐4), 8.62 (1H, d, ^*3*^ *J* = 8 Hz, H‐7), 9.25 (1H, d, ^*3*^ *J* = 8 Hz, H‐10), 9.27 (1H, d, ^*3*^ *J* = 8 Hz, H‐1), 10.40 (1H, s, H‐6).

^13^C‐NMR (DMSO‐d_6_, 150.1 MHz, δ (ppm)): 63.3 (CH_2_), 120.1 (CH‐4), 123.2 (C‐6a), 123.4 (CH‐10), 124.9 (CH‐1), 125.4 (C‐1a), 129.1 (C‐4ʹ), 130.4 (CH‐2), 130.6 (CH‐2ʹ), 130.7 (CH‐8), 132.1 (CH‐3), 132.2 (CH‐3ʹ), 132.7 (C‐1ʹ), 133.1 (CH‐7), 134.0 (C‐4a), 134.8 (C‐10a), 138.8 (CH‐9), 157.1 (CH‐6), 190.3 (CO).

^15^N‐NMR (DMSO‐d_6_, 60.8 MHz, δ (ppm)): 186.0.

HRMS (MALDI‐TOF/TOF) m/z: [M+H]^+^ Calcd for C_21_H_15_BrNO^+^ 378.0410; Found 378.0535.

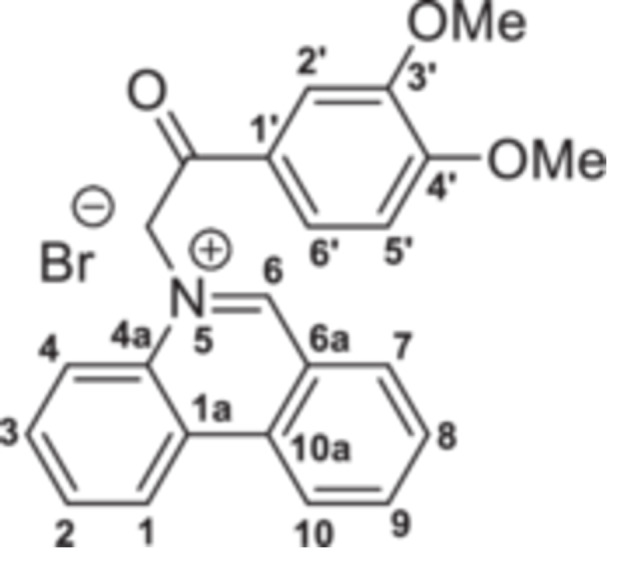

**5‐(2‐(3,4‐Dimethoxyphenyl)‐2‐oxoethyl)phenanthridin‐5‐ium bromide 3b:** White solid; η = 90%, mp = 217°C–218°C; IR(ATR) ν(cm^−1^): 3320, 3183, 3078, 2915, 1884, 2838, 2357, 2320, 1695, 1625, 1601, 1534, 1512, 1455, 1420, 1343, 1308, 1270, 1195, 1158, 1063, 1050, 990, 855, 837, 770, 752, 715, 678, 610, 579, 520, 490.

^1^H‐NMR (DMSO‐d_6_, 500.1 MHz, δ (ppm)): 3.88 (3H, s, CH_3_‐3’), 3.95 (3H, s, CH_3_‐4ʹ), 7.04 (2H, s, CH_2_), 7.30 (1H, d, ^*3*^ *J* = 9 Hz, H‐5’), 7.61 (1H, d, ^*4*^ *J* = 2 Hz, H‐2ʹ), 7.95 (1H, dd, ^*3*^ *J* = 9 Hz, ^*4*^ *J* = 2 Hz, H‐6ʹ), 8.11 (1H, t, ^*3*^ *J* = 8 Hz, H‐3), 8.14 (1H, d, ^*3*^ *J* = 8 Hz, H‐2), 8.18 (1H, t, ^*3*^ *J* = 8 Hz, H‐8), 8.40 (1H, d, ^*3*^ *J* = 8 Hz, H‐4), 8.51 (1H, t, ^*3*^ *J* = 8 Hz, H‐9), 8.63 (1H, d, ^*3*^ *J* = 8 Hz, H‐7), 9.25 (1H, d, ^*3*^ *J* = 8 Hz, H‐10), 9.27 (1H, d, ^*3*^ *J* = 8 Hz, H‐1), 10.34 (1H, s, H‐6).

^13^C‐NMR (DMSO‐d_6_, 150.1 MHz, δ (ppm)): 55.7 (CH_3_‐3ʹ), 56.1 (CH_3_‐4ʹ), 63.1 (CH_2_), 110.5 (CH‐2ʹ), 111.3 (CH‐5ʹ), 120.0 (CH‐4), 123.3 (C‐6a), 123.5 (CH‐10), 123.9 (CH‐6ʹ), 125.0 (CH‐1), 125.5 (C‐1a), 126.2 (C‐1ʹ), 130.4 (CH‐2), 130.7 (CH‐8), 132.2 (CH‐3), 133.1 (CH‐7), 134.0 (C‐4a), 134.7 (C‐10a), 138.8 (CH‐9), 148.9 (CO‐3ʹ), 154.5 (CO‐4ʹ), 157.1 (CH‐6), 189.1 (CO).

^15^N‐NMR (DMSO‐d_6_, 60.8 MHz, δ (ppm)): 186.8.

HRMS (MALDI‐TOF/TOF) m/z: [M+H]^+^ Calcd for C_23_H_20_NO_3_^+^ 359.1516; Found 359.1512.

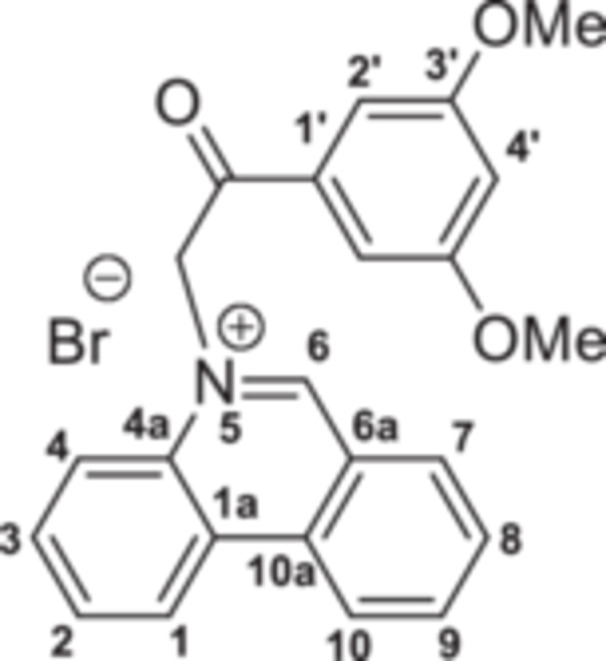

**5‐(2‐(3,5‐Dimethoxyphenyl)‐2‐oxoethyl)phenanthridin‐5‐ium bromide 3c:** White solid; η = 81%, mp = 216°C–217°C; IR(ATR) ν(cm^−1^): 3319, 3182, 3078, 2914, 1885, 2837, 2357, 2322, 1693, 1628, 1599, 1537, 1510, 1456, 1423, 1344, 1309, 1267, 1196, 1155, 1065, 1053, 989, 854, 837, 769, 750, 714, 677, 611, 577, 521, 490.

^1^H‐NMR (DMSO‐d_6_, 500.1 MHz, δ (ppm)): 3.89 (6H, s, CH_3_‐3ʹ), 6.99 (1H, t, ^*4*^ *J* = 2 Hz, H‐4’), 7.11 (2H, s, CH_2_), 7.34 (2H, d, ^*4*^ *J* = 2 Hz, H‐2ʹ), 8.11 (1H, t, ^*3*^ *J* = 8 Hz, H‐3), 8.14 (1H, t, ^*3*^ *J* = 8 Hz, H‐2), 8.17 (1H, t, ^*3*^ *J* = 8 Hz, H‐8), 8.44 (1H, d, ^*3*^ *J* = 8 Hz, H‐4), 8.50 (1H, t, ^*3*^ *J* = 8 Hz, H‐9), 8.62 (1H, d, ^*3*^ *J* = 8 Hz, H‐7), 9.25 (1H, d, ^*3*^ *J* = 8 Hz, H‐10), 9.27 (1H, d, ^*3*^ *J* = 8 Hz, H‐1), 10.39 (1H, s, H‐6).

^13^C‐NMR (DMSO‐d_6_, 150.1 MHz, δ (ppm)): 55.8 (CH_3_‐3ʹ), 63.5 (CH_2_), 106.4 (CH‐2ʹ), 106.5 (CH‐4’), 120.0 (CH‐4), 123.3 (C‐6a), 123.4 (CH‐10), 125.0 (CH‐1), 125.4 (C‐1a), 130.4 (CH‐2), 130.7 (CH‐8), 132.2 (CH‐3), 133.1 (CH‐7), 133.9 (C‐4a), 134.8 (C‐10a), 135.4 (C‐1’), 138.8 (CH‐9), 157.1 (CH‐6), 160.8 (C‐3ʹ), 190.7 (CO).

^15^N‐NMR (DMSO‐d_6_, 60.8 MHz, δ (ppm)): 186.7.

HRMS (MALDI‐TOF/TOF) m/z: [M+H]^+^ Calcd for C_23_H_20_NO_3_^+^ 359.1516; Found 359.1513.

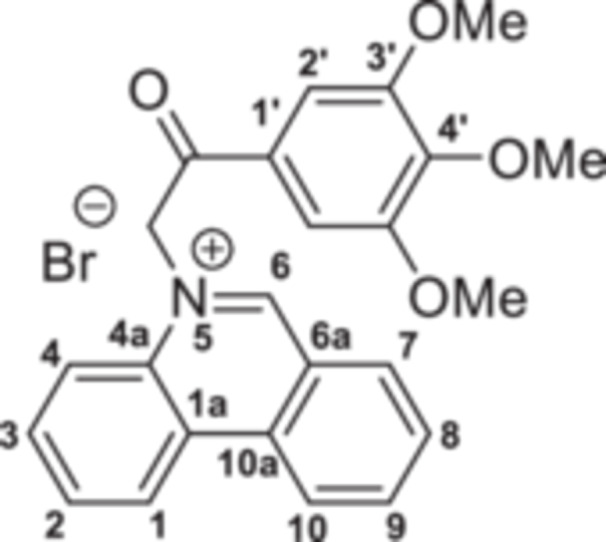

**5‐(2‐oxo‐2‐(3,4,5‐Trimethoxyphenyl)ethyl)phenanthridin‐5‐ium bromide 3d:** White solid; η = 95%, mp = 210°C–211°C; IR(ATR) ν(cm^−1^): 3325, 3180, 3080, 2915, 1880, 2840, 1699, 1628, 1600, 1538, 1515, 1455, 1427, 1340, 1310, 1272, 1198, 1160, 1065, 1059, 991, 855, 837, 777, 714, 680, 615, 580, 520, 490.

^1^H‐NMR (DMSO‐d_6_, 500.1 MHz, δ (ppm)): 3.84 (3H, s, CH_3_‐4ʹ), 3.94 (6H, s, CH_3_‐3ʹ), 7.11 (2H, s, CH_2_), 7.52 (2H, s, H‐2ʹ), 8.13 (1H, t, ^*3*^ *J* = 8 Hz, H‐3), 8.15 (1H, t, ^*3*^ *J* = 8 Hz, H‐2), 8.19 (1H, t, ^*3*^ *J* = 8 Hz, H‐8), 8.41 (1H, d, ^*3*^ *J* = 8 Hz, H‐4), 8.52 (1H, t, ^*3*^ *J* = 8 Hz, H‐9), 8.65 (1H, d, ^*3*^ *J* = 8 Hz, H‐7), 9.26 (1H, d, ^*3*^ *J* = 8 Hz, H‐10), 9.28 (1H, d, ^*3*^ *J* = 8 Hz, H‐1), 10.33 (1H, s, H‐6).

^13^C‐NMR (DMSO‐d_6_, 150.1 MHz, δ (ppm)): 56.3 (CH_3_‐3ʹ), 60.4 (CH_3_‐4ʹ), 63.3 (CH_2_), 106.3 (CH‐2ʹ), 119.9 (CH‐4), 123.3 (C‐6a), 123.5 (CH‐10), 125.0 (CH‐1), 125.5 (C‐1a), 128.6 (C‐1ʹ), 130.5 (CH‐2), 130.7 (CH‐8), 132.2 (CH‐3), 133.1 (CH‐7), 134.0 (C‐4a), 134.8 (C‐10a), 138.8 (CH‐9), 143.3 (C‐4ʹ), 153.0 (C‐3ʹ), 157.1 (CH‐6), 189.7 (CO).

^15^N‐NMR (DMSO‐d_6_, 60.8 MHz, δ (ppm)): 186.4.

HRMS (MALDI‐TOF/TOF) m/z: [M+H]^+^ Calcd for C_24_H_22_NO_4_^+^ 389.15433; Found 389.1586.

#### 4.1.2 General Procedure for the Preparation of Compounds **5a–d**

The cycloimmonium salt **3** (1 mmol) and ethyl propiolate (1.1 mmol) were added to dichloromethane (DCM) and the obtained suspension was stirred at room temperature. Then, a solution of triethylamine (TEA) (3 mmol) in DCM (3 mL) was added drop‐wise over 1 h (magnetic stirring) and the resulting mixture was then stirred overnight at room temperature. Methanol (10 mL) was added and the resulting solid was collected by filtration to give a solid which was washed with 5 mL methanol. The product was then crystallized from dichloromethane/methanol (1:1, v/v).

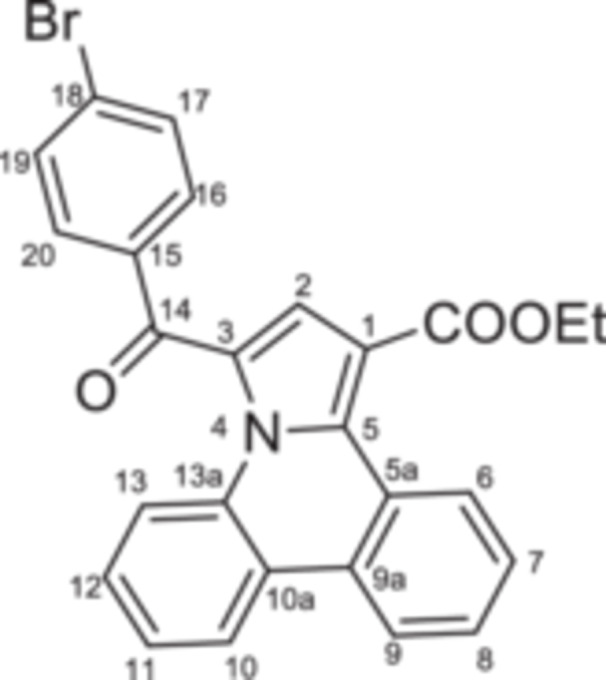

**Ethyl 3‐(4‐bromobenzoyl)pyrrolo[1,2‐** ***f*** **]phenanthridine‐1‐carboxylate 5a:** White solid; η = 73%, mp = 181°C–182°C; IR(ATR) ν(cm^−1^): 3222, 3128, 3070, 2976, 2902, 2357, 1774, 1712, 1645, 1581, 1525, 1500, 1450, 1439, 1394, 1373, 1322, 1275, 1234, 1196, 1171, 1138, 1120, 1093, 1069, 1034, 1009, 980, 903, 874, 845, 829, 758, 748, 721, 687, 631, 575, 521, 471, 459.

^1^H‐NMR (DMSO‐d_6_, 400.1 MHz, δ (ppm)): 1.34 (3H, t, ^*3*^ *J* = 7 Hz, CH_3_‐Et), 4.37 (2H, q, ^*3*^ *J* = 7 Hz, CH_2_‐Et), 7.55 (1H, t, ^*3*^ *J* = 7 Hz, H‐8), 7.56 (1H, s, H‐2), 7.60 (1H, t, ^*3*^ *J* = 7 Hz, H‐7), 7.68 (1H, dd, ^*3*^ *J* = 7 Hz, ^*4*^ *J* = 1.5 Hz, H‐9), 7.73 (1H, td, ^*3*^ *J* = 7 Hz, ^*4*^ *J* = 1.1 Hz, H‐11), 7.81 (1H, td, ^*3*^ *J* = 7 Hz, ^*4*^ *J* = 1.1 Hz, H‐12), 7.85 (2H, d, ^*3*^ *J* = 8 Hz, H‐17), 8.00 (2H, d, ^*3*^ *J* = 8 Hz, H‐16), 8.68 (1H, d, ^*3*^ *J* = 8 Hz, H‐6), 8.71 (1H, d, ^*3*^ *J* = 8 Hz, H‐13), 9.49 (1H, dd, ^*3*^ *J* = 8 Hz, ^*4*^ *J* = 1 Hz, H‐10).

^13^C‐NMR (DMSO‐d_6_, 100.6 MHz, δ (ppm)): 13.9 (CH_3_), 60.4 (CH_2_), 110.7 (C‐1), 121.3 (CH‐9), 122.2 (C‐10a), 122.8 (CH‐13), 123.2 (C‐3), 124.0 (CH‐6), 125.9 (CH‐7 and C‐5a), 127.3 (CH‐10), 127.8 (C‐18), 127.9 (C‐13a), 128.2 (CH‐11), 128.4 (CH‐8), 128.7 (CH‐2), 129.9 (CH‐12), 130.8 (C‐5), 131.5 (CH‐16), 131.7 (CH‐17), 136.2 (C‐15), 136.8 (C‐9a), 163.8 (COO), 183.2 (CO‐14).

^15^N‐NMR (DMSO‐d_6_, 60.8 MHz, δ (ppm)): 173.9.

HRMS (MALDI‐TOF/TOF) m/z: [M+H]^+^ Calcd for C_26_H_18_BrNO_3_ 472.0549; Found 472.0545.

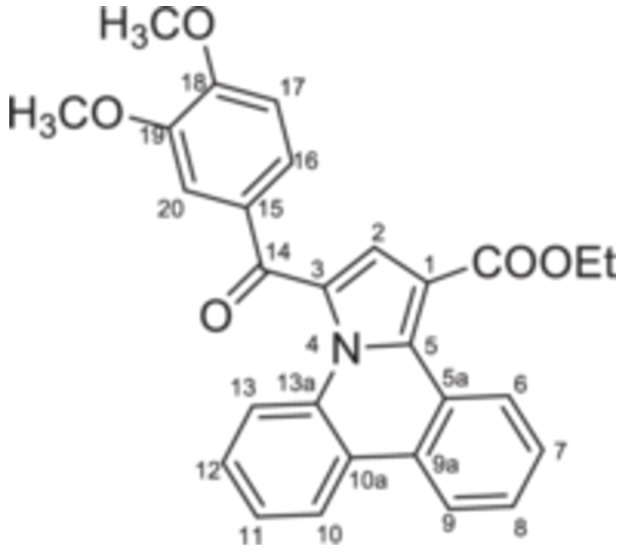

**Ethyl 3‐(3,4‐dimethoxybenzoyl)pyrrolo[1,2‐** ***f*** **]phenanthridine‐1‐carboxylate 5b:** White solid; η = 71%, mp = 188°C–189°C; IR(ATR) ν(cm^−1^): 3120, 3094, 2970, 2929, 2836, 1715, 1640, 1588, 1527, 1453, 1440, 1375, 1355, 1327, 1298, 1270, 1232, 1200, 1182, 1153, 1127, 1102, 1065, 1035, 1022, 945, 940, 925, 880, 845, 756, 742, 713, 615, 575, 503, 459, 426.

^1^H‐NMR (DMSO‐d_6_, 400.1 MHz, δ (ppm)): 1.35 (3H, t, ^*3*^ *J* = 7 Hz, CH_3_‐Et), 3.87 (3H, s, OCH_3_‐19), 3.92 (3H, s, OCH_3_‐18), 4.37 (2H, q, ^*3*^ *J* = 7 Hz, CH_2_‐Et), 7.20 (1H, d, ^3^ *J* = 8 Hz, H‐17), 7.52 (1H, s, H‐2), 7.55‐7.63 (4H, m, H‐8, H‐7, H‐9 and H‐20), 7.71–7.76 (2H, m, H‐11 and H‐16), 7.80 (1H, t, ^*3*^ *J* = 7 Hz, H‐12), 8.68 (1H, d, ^*3*^ *J* = 8 Hz, H‐6), 8.70 (1H, d, ^*3*^ *J* = 8 Hz, H‐13), 9.53 (1H, d, ^*3*^ *J* = 8 Hz, H‐10).

^13^C‐NMR (DMSO‐d_6_, 100.6 MHz, δ (ppm)): 13.9 (CH_3_), 55.6 (OCH_3_‐19), 55.7 (OCH_3_‐18), 60.3 (CH_2_), 110.3 (C‐1), 111.0 (CH‐17), 111.9 (CH‐20), 120.8 (CH‐9), 122.1 (C‐10a), 122.7 (CH‐13), 123.4 (C‐3), 124.0 (CH‐6), 124.8 (CH‐16), 125.6 (CH‐7), 127.0 (CH‐10), 127.1 (CH‐2), 127.5 (C‐13a), 128.1 (CH‐11), 128.3 (C‐5a), 128.4 (CH‐8), 129.4 (C‐15), 129.5 (CH‐12), 130.9 (C‐5), 135.9 (C‐9a), 148.8 (C‐19), 153.6 (C‐18), 163.9 (COO), 183.6 (CO‐14).

^15^N‐NMR (DMSO‐d_6_, 60.8 MHz, δ (ppm)): 174.1.

HRMS (MALDI‐TOF/TOF) m/z: [M+H]^+^ Calcd for C_28_H_23_NO_5_ 454.1655; Found 454.1657.

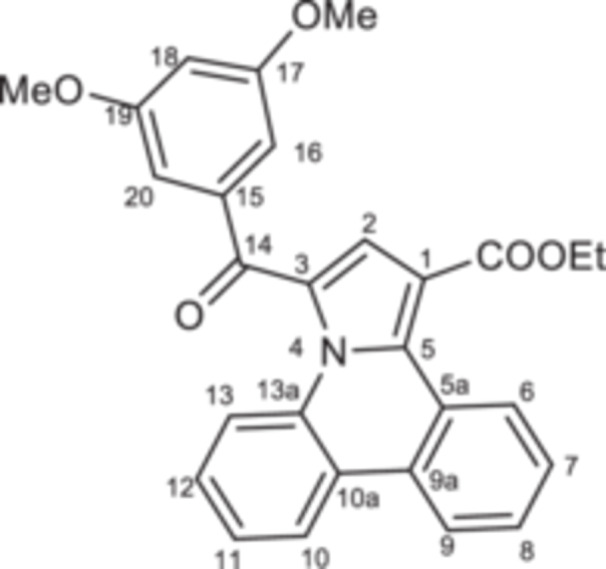

**Ethyl 3‐(3,5‐dimethoxybenzoyl)pyrrolo[1,2‐f]phenanthridine‐1‐carboxylate 5c:** White solid; η = 69%, mp = 187°C–188°C; IR(ATR) ν(cm^−1^): 3124, 3095, 2971, 2927, 2837, 1938, 1710, 1641, 1587, 1527, 1450, 1437, 1377, 1354, 1325, 1296, 1269, 1230, 1201, 1180, 1156, 1126, 1100, 1066, 1034, 1020, 949, 941, 924, 881, 845, 750, 742, 715, 615, 573, 503, 459, 426.

^1^H‐NMR (DMSO‐d_6_, 400.1 MHz, δ (ppm)): 1.35 (3H, t, ^*3*^ *J* = 7 Hz, CH_3_‐Et), 3.85 (6H, s, 2xOCH_3_), 4.37 (2H, q, ^*3*^ *J* = 7 Hz, CH_2_‐Et), 6.89 (1H, t, ^4^ *J* = 2 Hz, H‐18), 7.17 (2H, d, ^4^ *J* = 2 Hz, H‐16), 7.56–7.59 (2H, m, H‐2 and H‐8), 7.60 (1H, t, ^*3*^ *J* = 7 Hz, H‐7), 7.68 (1H, dd, ^*3*^ *J* = 7 Hz, ^*4*^ *J* = 1 Hz, H‐9), 7.73 (1H, td, ^*3*^ *J* = 7 Hz, ^*4*^ *J* = 1 Hz, H‐11), 7.80 (1H, td, ^*3*^ *J* = 7 Hz, ^*4*^ *J* = 1 Hz, H‐12), 8.68 (1H, d, ^*3*^ *J* = 8 Hz, H‐6), 8.71 (1H, d, ^*3*^ *J* = 8 Hz, H‐13), 9.51 (1H, d, ^*3*^ *J* = 8 Hz, H‐10).

^13^C‐NMR (DMSO‐d_6_, 100.6 MHz, δ (ppm)): 13.9 (CH_3_), 55.4 (OCH_3_), 60.4 (CH_2_), 105.1 (CH‐18), 107.5 (CH‐16), 110.5 (C‐1), 121.2 (CH‐9), 122.1 (C‐10a), 122.7 (CH‐13), 123.2 (C‐3), 124.0 (CH‐6), 125.8 (CH‐7), 127.2 (CH‐10), 127.8 (C‐13a), 128.1 (CH‐11), 128.2 (C‐5a), 128.3 (CH‐8), 128.5 (CH‐2), 129.7 (CH‐12), 130.8 (C‐5), 136.7 (C‐9a), 139.1 (C‐15), 160.4 (C‐17), 163.8 (COO), 183.7 (CO‐14).

^15^N‐NMR (DMSO‐d_6_, 60.8 MHz, δ (ppm)): 173.4.

HRMS (MALDI‐TOF/TOF) m/z: [M+H]^+^ Calcd for C_28_H_23_NO_5_ 454.1655; Found 454.1652.

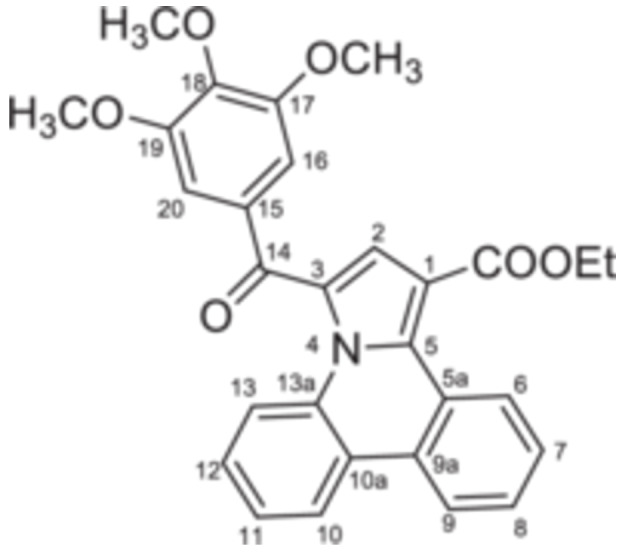

**Ethyl 3‐(3,4,5‐trimethoxybenzoyl)pyrrolo[1,2‐f]phenanthridine‐1‐carboxylate 5d:** White solid; η = 75%, mp = 176°C–177°C; IR(ATR) ν(cm^–1^): 3242, 3132, 3097, 2939, 2837, 2640, 2359, 2322, 2162, 1984, 1824, 1714, 1635, 1580, 1525, 1502, 1437, 1412, 1375, 1331, 1271, 1227, 1165, 1129, 1092, 1057, 1057, 997, 960, 922, 858, 816, 779, 754, 712, 677, 613, 569, 525, 432.

^1^H‐NMR (DMSO‐d_6_, 400.1 MHz, δ (ppm)): 1.35 (3H, t, ^*3*^ *J* = 7 Hz, CH_3_‐Et), 3.85 (3H, s, OCH_3_‐18), 3.87 (6H, s, OCH_3_‐17), 4.38 (2H, q, ^*3*^ *J* = 7 Hz, CH_2_‐Et), 7.40 (2H, s, H‐16), 7.55–7.61 (3H, m, H‐2, H‐8 and H‐7), 7.67 (1H, d, ^*3*^ *J* = 7 Hz, H‐9), 7.73 (1H, t, ^*3*^ *J* = 7 Hz, H‐11), 7.81 (1H, t, ^*3*^ *J* = 7 Hz, H‐12), 8.68 (1H, d, ^*3*^ *J* = 8 Hz, H‐6), 8.71 (1H, d, ^*3*^ *J* = 8 Hz, H‐13), 9.51 (1H, d, ^*3*^ *J* = 8 Hz, H‐10).

^13^C‐NMR (DMSO‐d_6_, 100.6 MHz, δ (ppm)): 13.9 (CH_3_), 56.1 (OCH_3_‐17), 60.1 (OCH_3_‐18), 60.3 (CH_2_), 107.7 (CH‐16), 110.5 (C‐1), 121.2 (CH‐9), 122.1 (C‐10a), 122.7 (CH‐13), 123.3 (C‐3), 124.0 (CH‐6), 125.6 (CH‐7), 127.1 (CH‐10), 127.7 (C‐13a), 127.9 (CH‐2), 128.1 (CH‐11), 128.2 (C‐5a), 128.3 (CH‐8), 129.6 (CH‐12), 130.8 (C‐5), 131.9 (C‐15), 136.4 (C‐9a), 142.4 (C‐18), 152.6 (C‐17), 163.9 (COO), 183.4 (CO‐14).

^15^N‐NMR (DMSO‐d_6_, 60.8 MHz, δ (ppm)): 173.7.

HRMS (MALDI‐TOF/TOF) m/z: [M+H]^+^ Calcd for C_29_H_25_NO_6_ 484.1761; Found 484.1757.

### 4.2 In Silico ADME and Toxicity Predictions

The ADME in silico evaluation for the compounds **3a–d** and **5a–d** was performed using the SwissADME web tool (http://swissadme.ch/index.php) in terms of molecular properties, pharmacokinetics, drug‐likeness, and medicinal chemistry.

The in silico toxicological evaluation for the most active compounds **3a–d** and **5a–d** was performed using the web service Cell‐Line Cytotoxicity Predictor (https://www.way2drug.com/clc-pred/), which screens for in silico cytotoxicity on a panel of 278 tumor cells and 27 normal human cell lines from different tissues.

### 4.3 Molecular Modeling

Molecular docking simulations were conducted with AutoDock 4.2.6 [33] to assess the binding behavior of the novel synthetized pyrrolo‐fused derivatives at the tubulin active site. The colchicine binding site is a well‐known target for anticancer drugs that bind to and block cell division [34]. The crystal structure of the tubulin‐colchicine complex was retrieved from the Protein Data Bank (PDB ID: 4O2B) [35] and used to extract the receptor model. Specifically, the α,β‐tubulin heterodimer, along with the existing GTP, GDP, and coordinated Mg²⁺ and Ca²⁺ ions, were retained in their original conformations. Besides those, to prepare the protein for docking, all water molecules, colchicine, and other co‐crystallized ligands or organic solvent molecules were removed. AutoDock Tools (ADT) v4.2 software [33] was used to add the polar hydrogens and to assign the atom types and Kollman charges. The protein was kept rigid during the docking simulations.

The ligand structures—Colchicine, Phenstatin, and the synthesized pyrrolo‐fused phenanthridine derivatives—were constructed using GaussView 5.0.9 [36] and subsequently optimized using DFT at the B3LYP/6‐31G level of theory with the Gaussian 16 software package.

A grid box of 60 × 60 × 60 points and a spacing of 0.375 Å was set around the geometric center of the colchicine‐binding pocket as defined by the position of colchicine in the original crystal structure (coordinates: x = 17.019, y = 65.993, z = 43.390), ensuring accurate coverage of the target site. Docking simulations were carried out using the Lamarckian Genetic Algorithm [37], which combines global stochastic search with local optimization. This approach enabled efficient exploration of ligand conformations and identification of energetically favorable binding modes within the target binding pocket. Each docking run generated 1000 poses per ligand, with a population size of 300 individuals, and a maximum number 2.5 × 10^6^ of energy evaluations. All poses were subsequently clustered and ranked by estimated binding energy. The estimated binding free energy (expressed in kcal/mol) is calculated by summing the contributions of van der Waals interactions, hydrogen bonding, desolvation energy, final total internal energy, and torsional free energy. From this total, a term for the unbound system energy is subtracted to yield the final binding energy estimate [37]. The lowest‐energy binding pose for each ligand was analyzed and the 3D representations were generated with PyMOL [38], and the 2D interaction diagrams were generated using Maestro [39].

### 4.4 In Vitro Cytotoxicity Assessment

Cytotoxicity of compounds was assessed on human gingival fibroblasts (HGF, population doubling time‐24 h), osteosarcoma (HOS, population doubling time‐36h, and MG‐63, population doubling time‐28 h) and malignant melanoma (MeWo, population doubling time‐32h, all from CLS Cell Lines Service GmbH, Eppelheim, Germany) using the CellTiter‐Glo 2.0 Assay (Promega, Madison, WI USA), according to the manufacturer's instructions. Cells were seeded into 96‐well opaque white tissue culture‐treated plates (50000 cells/mL) and allowed to adhere overnight in αMEM medium with 10% fetal bovine serum and 1% antibiotic‐antimycotic (all from Gibco, Thermo Fisher Scientific, Waltham, MA USA). Cells were incubated with compounds (10 or 50 µM) for 24 h, then CellTiter‐Glo reagent was added and luminescence was recorded using a FLUOstar Omega microplate reader (BMG LABTECH, Ortenberg, Germany). The experiments were carried out in triplicate, and the viability of treated cells was expressed as a percentage of the viability of control cells (untreated). Data were represented as means ± standard deviations.

## Author Contributions

The manuscript was written through the contributions of all authors. All authors have approved the final version of the manuscript. Synthesis was conducted by Ashraf Al‐Matarneh and Maria‐Cristina Al‐Matarneh; physical‐chemical characterization was made by Ashraf Al‐Matarneh, Maria‐Cristina Al‐Matarneh, Alina Nicolescu, Ramona Danac; theoretical prediction by Maria‐Cristina Al‐Matarneh, Ionel I. Mangalagiu, Narcis Cibotariu; cell‐based biological assays were conducted by Ashraf Al‐Matarneh, and Natalia Simionescu; conceptualization was made by Ionel I. Mangalagiu and Maria‐Cristina Al‐Matarneh.

## Conflicts of Interest

The authors declare no conflicts of interest.

## Supporting information

Supplementary Al‐Matarneh_et_al_manuscript.

## Data Availability

The data that support the findings of this study are available from the corresponding author upon reasonable request.
